# Tuberculosis-Associated Hospitalizations and Deaths after COVID-19 Shelter-In-Place, San Francisco, California, USA

**DOI:** 10.3201/eid2708.210670

**Published:** 2021-08

**Authors:** Janice K. Louie, Rocio Agraz-Lara, Laura Romo, Felix Crespin, Lisa Chen, Susannah Graves

**Affiliations:** San Francisco Department of Public Health Tuberculosis Prevention and Control Program, San Francisco, California, USA (J.K. Louie, R. Agraz-Lara, L. Romo, F. Crespin, S. Graves);; University of California, San Francisco (J.K. Louie, L. Chen)

**Keywords:** tuberculosis and other mycobacteria, TB, coronavirus disease, COVID-19, SARS-CoV-2, severe acute respiratory syndrome coronavirus 2, epidemiology, hospitalizations, mortality rate, deaths, bacteria, viruses, respiratory diseases, respiratory infections, zoonoses, San Francisco, California, United States

## Abstract

A mandated shelter-in-place and other restrictions associated with the coronavirus disease pandemic precipitated a decline in tuberculosis diagnoses in San Francisco, California, USA. Several months into the pandemic, severe illness resulting in hospitalization or death increased compared with prepandemic levels, warranting heightened vigilance for tuberculosis in at-risk populations.

## Abstract 2

After an initial decline, tuberculosis cases involving severe illness increased.

Since the emergence of a novel coronavirus, severe acute respiratory syndrome coronavirus 2 (SARS-CoV-2), which causes coronavirus disease (COVID-19), unprecedented measures have been recommended to reduce transmission. In San Francisco, California, USA, progressively restrictive health officer orders implemented since early 2020 have included travel quarantines, shelter-in-place (SIP), deferral of routine medical appointments and elective surgeries, closure of public-facing events and businesses, and isolation and quarantine when appropriate (*1*). Nationwide, disruptions in medical services have contributed to delaying or avoiding routine care and a decrease in non–COVID-19-related hospital admissions and emergency department visits (*2*). Similarly, worldwide tuberculosis (TB) case reports have declined, including in San Francisco, where a ≈60% decrease in newly diagnosed TB cases compared with prior years was observed in the first 4 months of the pandemic (*3*,*4*).

The San Francisco Department of Public Health (SFDPH) Tuberculosis Prevention and Control Program manages all cases of active TB in San Francisco residents (≈881,549 population). In 2019, San Francisco had a high incidence of TB, with rates >4-fold higher (11.9 cases/100,000 persons) than the national rate. The affected population is predominantly non–US-born (86%) with >80% residing in the United States >5 years (*5*). We reviewed overall numbers of active TB case-patients in San Francisco and newly diagnosed cases including those resulting in hospitalization, intensive care unit admission, and death. We divided our analysis into 2 periods: pre-SIP (January 1, 2019–March 15, 2020) and during SIP (March 16, 2020–January 31, 2021). TB was reportable within 1 working day of diagnosis. Cases were diagnosed by microbiologic testing or medical assessment for consistent clinical and radiographic findings. All patients who received a TB diagnosis after SIP began were tested for SARS-CoV-2 co-infection at the time of TB diagnosis, except for 7 patients during March–May 2020, when testing was less available. For all fatalities, we used a standardized algorithm to review medical records and death certificates to determine whether cause of death was TB-related. Because these activities were public health surveillance and not research, review by institutional review board was not requested.

During the 14.5-month pre-SIP period, the monthly average number of patients receiving TB treatment was 73.0 persons, compared with 42.7 persons during the 10.5-month SIP period, resulting in a 42% reduction. The initial SIP period was marked by low numbers of new TB diagnoses during mid-March through June; increasing numbers starting in July, when more case-patients had TB diagnosed while they were hospitalized or dying from TB (Figure). Pre-SIP, a total of 114 patients (average 7.9 patients/month) were newly diagnosed with TB. A total of 38 (33.3%) patients were hospitalized, including 5 (4.4%) who required intensive care. A total of 4 (3.5%) patients died with cause of death assessed as TB-related. In comparison, after SIP began, 52 patients (average 5.0 patients/month) were newly diagnosed with TB. A total of 33 (63.5%) patients were hospitalized, including 12 (23.1%) patients who required intensive care; 7 (13.5%) patients died with cause of death assessed as TB-related. No patients diagnosed with TB during SIP reported having previous SARS-CoV-2 infection; all patients screened for SARS-CoV-2 had negative results. One patient experienced new-onset low-grade fever and cough 37 days after starting TB treatment and subsequently tested SARS-CoV-2 positive; this patient had no new radiographic abnormalities or COVID-19–related complications. More patients during SIP than before SIP required hospitalization, received intensive care, or had a TB-related death (p<0.05 by Pearson χ^2^ test (Table). We found no difference in duration of TB symptoms pre-SIP (median 1 month, range 0–120 months) than that during SIP (median 1.5 months, range 0–24 months).

**Figure:**
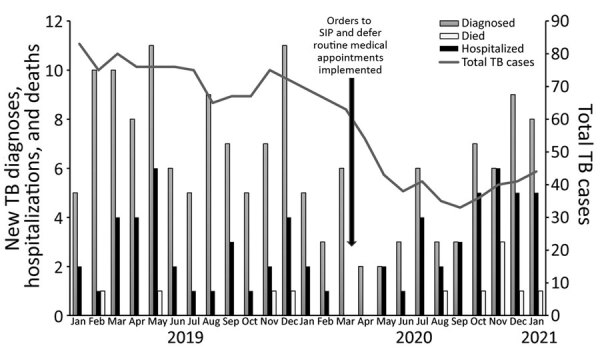
Comparison of TB cases pre-SIP (January 1, 2019–March 15, 2020) and after SIP (March 16, 2020–January 31, 2021), San Francisco, California, USA. Scales for the *y*-axes differ substantially to underscore patterns but do not permit direct comparisons. Total TB cases indicates total number of case-patients receiving TB treatment, by month. Cases were counted according to the month when TB was diagnosed. In the first months of the pandemic after SIP was implemented (March 16–June 30, 2020), numbers of patients newly diagnosed with TB decreased compared with the 14.5 months prior. In early July 2020, the number of patients newly diagnosed with TB began to increase, with a higher proportion requiring hospitalization or having a TB-related death. SIP, shelter-in-place; TB, tuberculosis.

**Table:** New diagnoses of TB pre-SIP compared with during SIP during the coronavirus disease pandemic, San Francisco, California, USA, January 1, 2020–January 30, 2021*

Variable	Pre-SIP: 2019 Jan 1–2020 Mar 15	SIP: 2020 Mar 16–2021 Jan 30	p value†
New diagnoses of active TB	114 (100)	52 (100)‡	NA
Average no. new TB cases/month	7.9	5.0	NA
Median age of case-patients, y (range)	64.0 (3–101)	66.0 (15–97)	NS
New case-patients with cavitary TB	33 (28.9)	11 (21.2)	NS
New TB case-patients requiring hospitalization	38 (33.3)	33 (63.5)	0.0003
New TB case-patients requiring intensive care	5 (4.4)	12 (23.1)	0.0002
New TB case-patients who died	15 (13.2)	10 (19.2)	NS
New TB case-patients with TB-related deaths§	4 (3.5)	7 (13.5)	0.017

*Values are no. (%) unless indicated. Cases were counted according to the month when TB was diagnosed. NA, not applicable; NS, not significant (p>0.05); SIP, shelter-in-place; TB, tuberculosis. †By Pearson χ^2^ test. ‡All patients were tested for severe acute respiratory syndrome coronavirus 2 co-infection at time of TB diagnosis, except for 7 patients for whom testing was not routinely available in the first 3 months of the pandemic (March–May 2020); no patients were positive. §Cause of death was evaluated by standardized algorithm and review of medical records and death certificate, when available. Deaths were counted as TB-related when TB was assessed as an immediate or contributing cause of death.

Our preliminary findings suggest that delayed TB diagnosis early in the pandemic, coinciding with implementation of SIP and other restrictive measures, might have contributed to an increasing proportion of patients who later experienced severe illness or death. Although we used SIP as a proxy, other factors probably contributed to the trend. The same racial, ethnic, and socioeconomic inequities that contributed to limited healthcare access during the COVID-19 pandemic are prevalent in TB-infected populations (*6*). Symptomatic patients might have been reluctant or unable to seek medical evaluation, thereby leading to worsening TB disease. The overlap of signs, symptoms, and abnormal radiographic findings for COVID-19 and TB could have resulted in prioritizing COVID-19 screening over TB diagnosis.

Our observations are a snapshot in time and are not representative of TB activity in other cities or regions where COVID-19 transmission rates and corresponding SIP and public health responses differ. Nevertheless, we collected real-world data consistent with the Stop-TB Partnership modeling studies predicting that prolonged disruption of TB activities could result in an excess of millions of TB cases and deaths through 2025 (*7*). As vaccination rates increase and restrictions ease, continued vigilance and public messaging about the importance of early diagnosis of TB in high-risk populations remain critical.
